# Understanding the trade-off between the environment and fertility in cows and ewes

**DOI:** 10.1590/1984-3143-AR2020-0017

**Published:** 2020-08-21

**Authors:** Hilary Dobson, Jean Elsie Routly, Robert Frank Smith

**Affiliations:** 1 Institute of Veterinary Science, University of Liverpool, Leahurst Campus, Neston, England

**Keywords:** oestrus, adrenal, GnRH, neurotransmitters, behaviour

## Abstract

The environment contributes to production diseases that in turn badly affect cow performance, fertility and culling. Oestrus intensity is lower in lame cows, and in all cows 26% potential oestrus events are not expressed (to avoid getting pregnant). To understand these trade-offs, we need to know how animals react to their environment and how the environment influences hypothalamus-pituitary-adrenal axis (HPA) interactions with the hypothalamus-pituitary-ovarian axis (HPO). Neurotransmitters control secretion of GnRH into hypophyseal portal blood. GnRH/LH pulse amplitude and frequency drive oestradiol production, culminating in oestrus behaviour and a precisely-timed GnRH/LH surge, all of which are disrupted by poor environments. Responses to peripheral neuronal agents give clues about mechanisms, but do these drugs alter perception of stimuli, or suppress consequent responses? *In vitro* studies confirm some neuronal interactions between the HPA and HPO; and immuno-histochemistry clarifies the location and sequence of inter-neurone activity within the brain. In both species, exogenous corticoids, ACTH and/or CRH act at the pituitary (reduce LH release by GnRH), and hypothalamus (lower GnRH pulse frequency and delay surge release). This requires inter-neurones as GnRH cells do not have receptors for HPA compounds. There are two (simultaneous, therefore fail-safe?) pathways for CRH suppression of GnRH release via CRH-Receptors: one being the regulation of kisspeptin/dynorphin and other cell types in the hypothalamus, and the other being the direct contact between CRH and GnRH cell terminals in the median eminence. When we domesticate animals, we must provide the best possible environment otherwise animals trade-off with lower production, less intense oestrus behaviour, and impaired fertility. Avoiding life-time peri-parturient problems by managing persistent lactations in cows may be a worthy trade-off on both welfare and economic terms – better than the camouflage use of drugs/hormones/feed additives/intricate technologies? In the long term, getting animals and environment in a more harmonious balance is the ultimate strategy.

## ‘Random’ observations to put ‘trade-offs’ in perspective

First: over a 2–3 week period, more than 80% non-domesticated wildebeest roaming the plains of the Serengeti in east Africa mate and calve with no interference by man (Estes, 1966). The trade-off? Predation of neonates by other animals is limited during synchronised births due to the formation of protective mothering groups and crèches, also aided by the extraordinary mobility of precocious calves. However, this contrasts with life expectancy as sick animals do not survive to breed.

**Figure 1:**
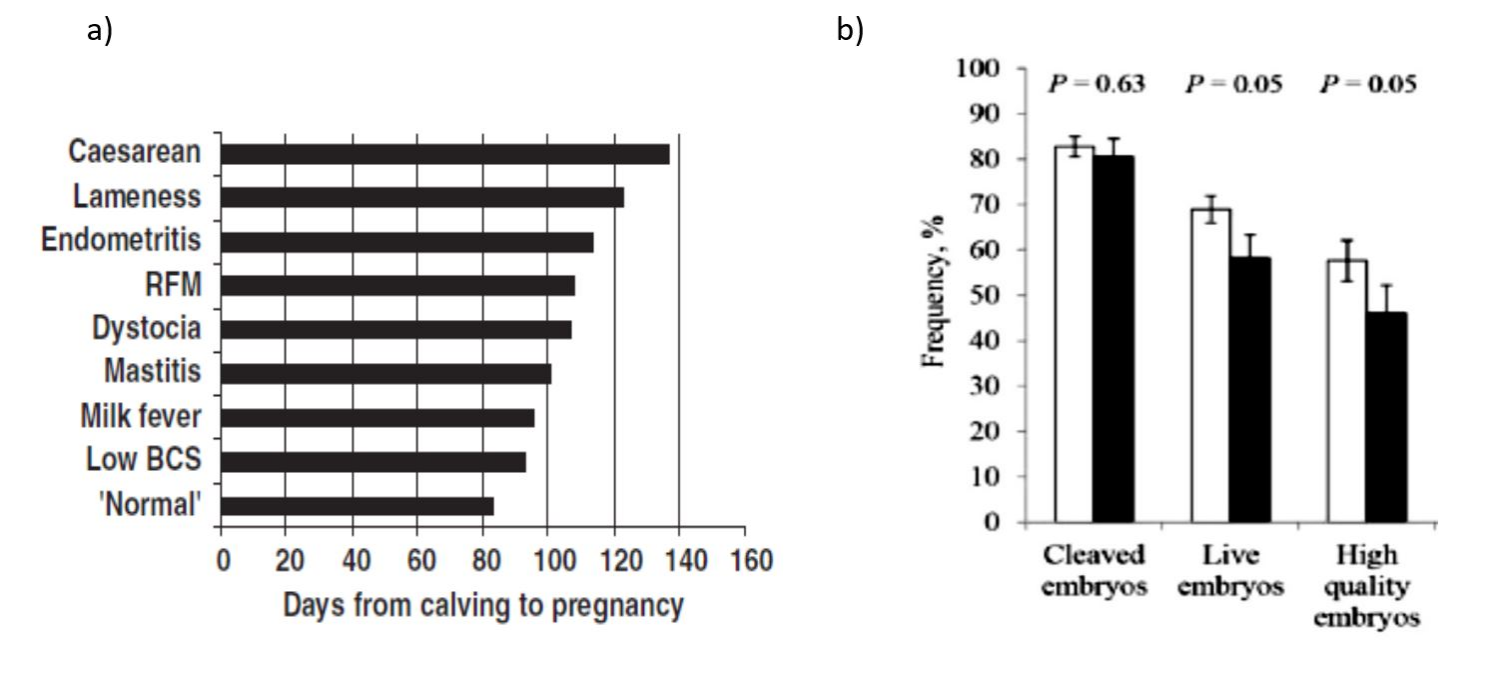
(a) Days from calving to pregnancy after AI in cows with different clinical production diseases (RFM retained fetal membranes; BCS body condition score). Adapted from Dobson et al. (2008); reproduced with permission; (b) Mean percentage of total embryos recovered (±SEM) representing cleaved embryos, live embryos (quality Grades 1-3) and high quality embryos (Grades 1 and 2) obtained from donors without (open bars) or with prior non-uterine disease (black bar). Adapted from Ribeiro et al. (2016); reproduced with permission.

Second: in dairy cows, production diseases predominate in the first 30 days after calving. Uterine problems represent major detrimental direct effects of the environment on fertility that are long-lasting (Sheldon and Owens, 2017; Piersanti et al., 2019); however, the present review focuses on non-uterine conditions. Such production diseases have an incidence of 6.7% ovarian cysts, 15.2% lameness, 17.2% subclinical ketosis; and treated cows take longer to get pregnant, 64, 100 and 58 days, respectively, (Figure 1a; Borsberry and Dobson, 1989; Collick et al., 1989; Rutherford et al., 2016). Milk fever also extends the calving to pregnancy interval by 12 days (Dobson et al., 2001), and cows with mastitis around the time of the first ‘silent’ oestrus have delayed luteal activity and late onset of oestrus (Huszenicza et al., 2005). So unsurprisingly, these conditions occur in 60-80% repeat breeders (≥ 3 unsuccessful inseminations; Canu et al., 2010). Indeed, non-uterine inflammatory diseases (mastitis, lameness, digestive and respiratory problems occurring before breeding) reduce rates of oocyte fertilization and development to morulae, and impair both elongation of early conceptuses and secretion of interferon-τ in the uterine lumen. Furthermore, these diseases cause changes in the transcriptome of conceptus cells, increase the risk of pregnancy loss, and reduce pregnancy or calving per breeding (Figure 1b; Ribeiro et al., 2016). Thus, treated production diseases have long-term effects on milking performance, fertility and culling of dairy cows, all of which are detrimental to the sustainability of dairy herds (Carvalho et al., 2019). Is this the price to be paid in the trade-off during domestication for milk production?

Third: the environment is often compromised during domestication. Adequate housing is important for cow comfort, but while straw-yards increase dairy cow lying time, a trade-off is made against an increase in mastitis (Whitaker et al., 2000). In cubicles with a sand-base, the prevalence of lameness is halved compared to mattresses, and cows spend more time eating (Cook et al., 2004). In addition, cows housed on slippery walk-ways express less intense oestrus with a major impact on pregnancy rates to AI (Britt et al., 1986). Also, high environmental temperatures (>25 ^0^C, either within housing, or outside without shade) reduce fertility, and this is even more dramatic for high yielding cows that also generate more heat than they can dissipate (Al Katanani et al., 1999). A useful management tool to maintain high pregnancy rates throughout the year would be to produce bovine embryos during the cooler months and use them in embryo transfer during periods of heat stress (Bo et al., 2019). Thus, providing more expensive environments or engaging intricate technologies (e.g., embryo production/transfer) are used in trade-offs against the deleterious effects of the environment on fertility (review: Dobson et al., 2001; review: Butler, 2003; Canu et al., 2010).

Fourth: for many years dairy farmers have been criticised for increasing milk yield per cow, while not spending enough time to observe oestrus properly, or even resorting to fixed-time inseminations. Also, herd size has increased in an attempt to produce more milk per farm – a trade-off between economies of scale against a reduction in attention-to-detail on an individual cow basis. Would the use of motion sensors (pedometers or neck collars) improve oestrus detection? When progesterone profiles identify possible oestrus events (periods of low milk progesterone with higher values before and after), motion sensors and observations by farm staff detect only 74% potential oestrus events (Holman et al., 2011; Williams et al., 2018). So, why do cows not show signs of oestrus in the remaining 26% situations – a trade-off to avoid getting pregnant again?

To understand these trade-offs, it is necessary to know how animals react to their domesticated environment (i.e., all pressures: production diseases, milk/meat yield, housing, feed, social interactions) and how cows/ewes translate their responses in terms of controlling fertility. In large part, this involves hormonal control of the hypothalamus-pituitary-adrenal axis (HPA) and its interaction with the hypothalamus-pituitary-ovarian axis (HPO). To examine these relationships, all our studies (including clinical field work) are carried out under UK Home Office licenses for work on living animals and with the approval of the University of Liverpool Ethical Review process.

## Summary of ovarian follicular phase endocrinology (HPO)

In many of the above ‘trade-off’ situations, either luteinising hormone (LH) pulse or surge patterns (or both) in dairy cows are disrupted leading to reduced fertility (feeding: Butler, 2003; environmental temperature: Badinga et al., 1994; mastitis: Hockett et al., 2005; lameness: review: Dobson et al., 2008). Greater knowledge about the control of oestrus cycle hormones (including LH) will lead to a better understanding of how animals optimise fertility, and how disruption occurs.

**Figure 2:**
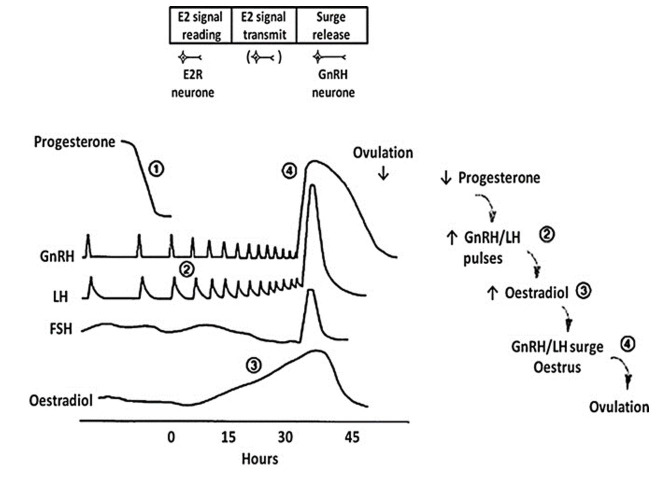
Endocrine events during the follicular phase of the oestrous cycle of ewes. Left, peripheral hormone patterns; right, sequence of regulatory steps; top, theoretical model of neuroendocrine processes involved in generating the GnRH/LH surge (timings as in lower panel). The numbers (1-4) represent steps for which there is evidence of interruption during adverse environmental stimuli (see text). Adapted from Battaglia et al. (1998, 2000) and Dobson et al. (2012); reproduced with permission.

Briefly, in cows and ewes neurotransmitters in the brain (especially hypothalamus) control secretion of gonadotrophin releasing hormone (GnRH) into hypophyseal portal blood, and thus LH release from the pituitary into the peripheral circulation. During the luteal phase, feedback from progesterone and oestradiol restrain small discrete GnRH/LH pulses to approximately one per four hours. As peripheral progesterone concentrations decline, LH pulse amplitude and frequency increase to approximately one per hour further driving ovarian follicular growth and oestradiol production. Towards the end of the follicular phase, when progesterone concentrations are low and oestradiol concentrations are at a maximum (Figure 2; oestradiol-signal reading phase; Battaglia et al., 2000), there is a temporary decrease in GnRH/LH pulse frequency and amplitude at approximately 4–6 h (oestradiol signal transmission phase) before a precisely-timed pre-ovulatory surge release of GnRH/LH. This causes ovulation and formation of a corpus luteum. Alternating exposure to oestradiol and progesterone leads to the expression of sexual behaviour and prepares the uterine environment; and in concert with signals from the conceptus, pregnancy will be established.

If the environment is not ideal, in cows and ewes GnRH/LH pulsatility is disrupted, as well as the timing and amplitude of the GnRH/LH surge, resulting in failure to initiate a pregnancy. This key disruption is usually temporary so that when prevailing conditions improve, normal hormonal profiles will resume.

## The environment - nutrition, milk yield and ovarian cysts in dairy cows

A major difficulty encountered by high-yielding dairy cows is achieving sufficient food dry matter intake (DMI) around calving. Feeding in the dry period has very important consequences for events in the following postpartum period. For example, in the week before calving, feeding duration and DMI are both more than 20% lower in cows that subsequently have mild-or-severe metritis and these same cows produce 6-8 kg milk/day less than healthy herd-mates during the first three weeks after calving (Huzzey et al., 2007). Low DMI is also associated with poor body condition scores and delayed return to normal ovarian cyclicity just after calving; a marked improvement in pregnancy rates occurs once DMI increases resulting in positive energy balance (review: Butler, 2003). Another example of a trade-off – poorly fed animals will switch off reproductive function until their nutritional needs are met.

A further example regarding the consequences of the genetic drive for higher yields: patterns of monthly milk yield and maximum values are not different from normal in those cows that develop ovarian cysts, but weekly analysis reveals shorter durations of peak yield (Nanda et al., 1989a). It is not certain whether this is cause or effect, but some cows trying to meet requirements of sustained milk yields are on a ‘knife-edge’ and more susceptible to minor environmental changes (such as new social interactions, or changes in diet; Dobson and Smith, 1998). High milk yield may just be one factor leading to formation of ovarian cysts, but there is no correlation between the incidence of cysts with either 3-beta-hydroxy-butyrate (BHB) concentration or body condition scores (Dobson and Nanda, 1992; Tebble et al., 2001). However, these may not be the best indicators: Jackson et al. (2011) provide evidence that high non-esterified fatty acid (NEFA) values and low urea:BHB ratios can be used before calving as predictors of an increased risk of endometritis, whereas high *Nu* values (multiplication of NEFA and urea values) and low urea:BHB ratios are more useful after calving as predictors of an increased likelihood of cystic ovaries and delayed commencement of luteal activity.

Prolonged anoestrus after calving is characterised by low oestradiol and progesterone values due to suppression of GnRH/LH pulsatility (review: Butler, 2003). Whereas follicular cysts (thin-walled oestradiol-producing structures > 2.5 cm diameter) form after failure of a timely GnRH/LH surge; although several days later sufficient LH may be secreted to produce a luteal cyst (progesterone- producing, > 2.5 cm diameter). Evidence for this suggestion follows:

Dairy cows with naturally-occurring follicular cysts have lower plasma progesterone concentrations than those with luteal cysts (Dobson et al., 1977); but cows with luteal cysts have more additional follicles > 5 mm diameter. Cows with both follicular cysts and other follicles > 5 mm diameter have oestradiol concentrations of ~ 8 pg/ml compared to ~24 pg/ml in cows without other follicles > 5 mm in diameter on either ovary (Douthwaite and Dobson, 2000). It is of note that Nanda et al. (1991a) found that half the cows with follicular cysts, and half the cows with luteal cysts (after prior prostaglandin treatment) respond with an LH surge after an oestradiol injection; therefore, both types of cyst could be due to defects in the LH surge mechanism.

As it is impossible to closely monitor cows at the precise moment ovarian cysts are spontaneously formed, experimental models have been developed. Starting on Day 15 of a ‘normal’ oestrus cycle, in heifers receiving either:

- high doses of oestradiol and progesterone to mimic high values at the end of pregnancy; 3 out of 8 formed persistent follicles (Ward et al., 2000);
- repeated doses of ACTH to stimulate adrenal secretion; 6 out of 13 formed persistent follicles (Dobson et al., 2000a);
- or prolonged low doses of progesterone to replicate low progesterone values seen after adrenal stimulation; all 13 formed persistent follicles (Noble et al., 2000).

Combined observations from these models reveal that during treatment

**Figure 3:**
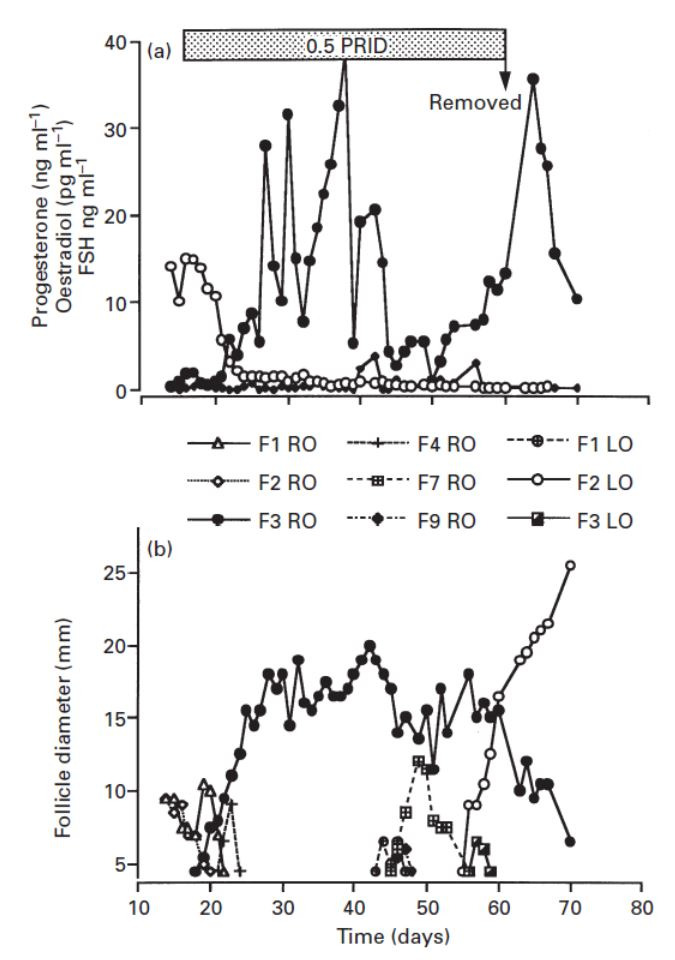
Daily plasma concentrations of (a) progesterone (○) and oestradiol (●), and (b) internal diameters of dominant follicles (●, ○) and subordinate follicles (other symbols) in cows from the last observed oestrus. The horizontal bar indicates presence of half a progesterone releasing intravaginal device (0.5 PRID). RO: right ovary, LO: left ovary. Note the presence of dominant follicle (●) for 50 days, but functionally producing oestradiol for only ~ 25 days; then replaced by a second functional dominant follicle (○) from 55 days onward. Adapted from Noble et al. (2000); reproduced with permission.
- subsequent treatment with very low dose progesterone or GnRH decreases plasma oestradiol and a new follicular wave emerges.

- follicle stimulating hormone (FSH) concentrations are normal;
- LH pulse and surge concentrations are lower;
- some prolonged dominant follicles ovulate after 10 days with higher oestradiol concentrations and greater internal diameters than those follicles that persist for > 20 days;
- occasionally persistent follicles luteinise;
- for the second half of the life-span of persistent follicles, oestradiol values are basal, thus the structure can remain for up to 50 days after endocrine function declines, and other smaller follicles appear (Figure 3);

In short, heifers with experimentally-produced persistent follicles resemble cows with clinically-diagnosed spontaneous cysts. But questions remain – why/how are pulsatile and surge LH secretion disrupted?

**Figure 4:**
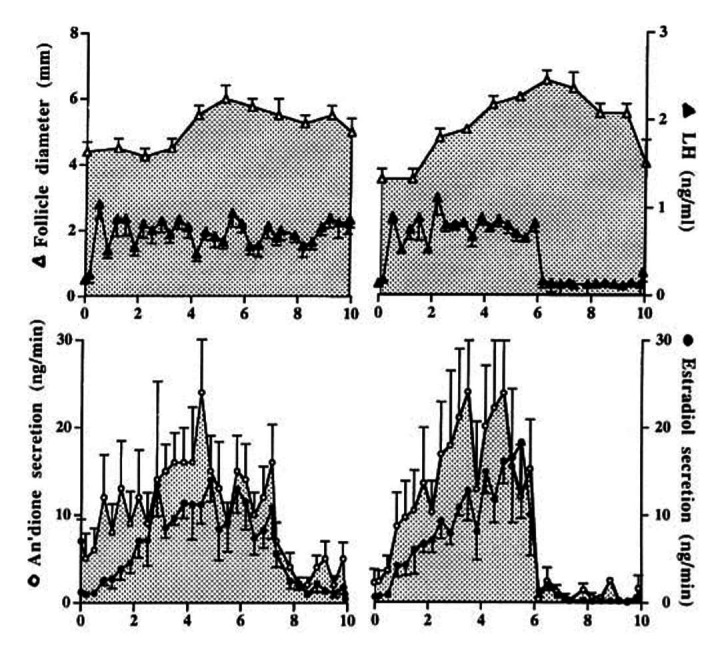
Mean (± SEM) diameter of the largest follicle, peripheral plasma concentrations of LH, and ovarian secretion rates of androstenedione and oestradiol, in ewes treated with GnRH antagonist and hourly injections of LH. The latter were continued for 10 days or stopped on Day 6 (right panels). Adapted from Dobson et al. (1997); reproduced with permission.

Ewe models have also been used to examine in greater detail the functionality, and consequences, of persistent follicles. In ewes, endogenous LH is suppressed by a GnRH antagonist but when replaced with frequent injections of low dose LH for 60 h, normal follicular growth occurs with ovulation after a large LH injection at 60 h, followed by a luteal pattern of plasma progesterone (Campbell et al., 1997). However, if the low dose injections of LH continue for 10 days (with no ovulatory dose of exogenous LH), follicular growth and oestradiol secretion continues for 8 days but then follicular atresia occurs (due to changes within the follicle). Alternatively, stopping the low dose LH injections after 4 days results in an immediate decrease in LH concentrations with a consequent decline in oestradiol secretion but large non-functional follicle structures remain (Figure 4; Dobson et al., 1997). Similarly, if the frequency of low dose LH injections is reduced at 30 h from hourly to as little as every 2 h, there is a marked reduction in oestradiol secretion (review: Dobson and Smith, 1998).

Regarding the consequences of prolonged oestradiol exposure, ovariectomised ewes treated with oestradiol implants for 2 to 12 days do not have a normal LH surge after a final challenge with oestradiol (Ozturk et al., 1998). However, LH secretion is provoked by repeated low dose GnRH injections, and, although responses are reduced by 50%, a lowered self-priming effect is still evident indicating inhibitory effects occur at both hypothalamic and pituitary level (Ozturk et al., 1998). By using physiological concentrations, these observations show that it is the duration of oestradiol exposure that causes problems, not excessive concentrations. Furthermore, correction of this oestradiol-induced lesion by administration of physiological does of progesterone for 12 days strengthens the previously empirical choice of similar treatment of clinical bovine cases with follicular cysts (Nanda et al., 1989a; Douthwaite and Dobson, 2000). Progesterone can be directly administered, or endogenous concentrations induced by GnRH treatment in cows (Ribadu et al., 1994).

Clearly, in cows and ewes continuation of LH pulses are required for persistent (cystic) follicular growth and oestradiol production, but why does an LH surge not occur at the end of what appears to be an otherwise normal follicular phase? Cystic follicles in cows are associated with clinical production diseases, including uterine infection (Tsousis et al., 2009). A better understanding of the HPA (and interaction with the HPO) may explain why/how production diseases result in the failure of LH surges during this trade-off.

## Summary of adrenal gland endocrinology (HPA)

**Figure 5:**
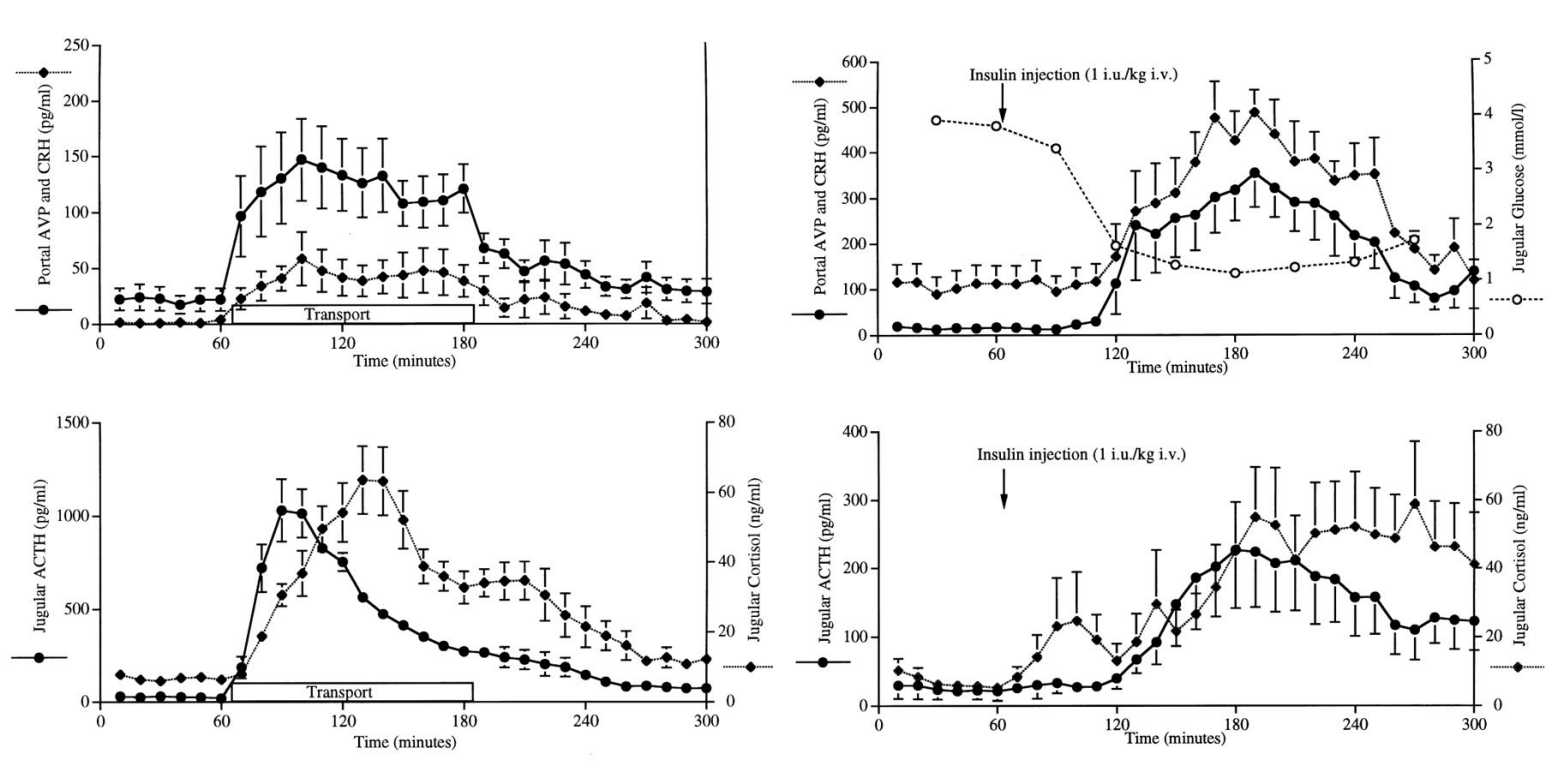
Ewe hypothalamus-pituitary-adrenal responses (mean ± SEM) to 2 h transport (left panels) or insulin injection (right panels). Note different vertical axis between left and right panels; inverse proportions of AVP and CRH after transport or insulin; and decreases in ACTH and cortisol while the stimuli continue. Adapted from Dobson and Smith (2000b); reproduced with permission.

The hypothalamus-pituitary-adrenal axis (HPA) protects life by monitoring the environment and activating immediate responses to threatening stimuli. Responses are similar in all mammals; during transport, both corticotrophin releasing hormone (CRH) and vasopressin (AVP) are released from the hypothalamus, which in turn cause the anterior pituitary to secrete adreno-corticotrophin hormone (ACTH); that then triggers the adrenal glands to secrete corticoids (predominantly cortisol in cows and ewes; Figure 5; Dobson and Smith 2000b, Smith et al., 2003a, b), as well as transient small amounts of progesterone. Different stimuli differ in intensity as judged by varying concentrations and durations of cortisol and progesterone in peripheral plasma (Smith et al., 2003a; Fergani et al., 2012). Psycho-social stimuli (social isolation, restraint, blindfolding and exposure to predatory cues), transport or rapidly lowered glucose values (after insulin injection), all result in lower cortisol profiles than administration of lipopolysaccharide toxin from *E coli* (LPS). However, there are several important points to note:-

- Maximum ACTH and cortisol values increase rapidly in ewes after the onset of most stimuli but ACTH and cortisol decreases occur very quickly with negative-feedback at both the hypothalamus and pituitary (Figure 5; Smith et al., 2003a, b).
- Chronically lame cows do **not** have continually elevated concentrations of cortisol (Walker et al., 2008a).
- Lower cortisol responses occur during the last of a series of transport stimuli in ewes (Smith and Dobson, 2001), i.e., there is an adaptation of cortisol responses to stimuli, due to individual perceptions, experience and/or responses.
- Acute stimuli immediately release adrenaline from the adrenal glands (Parrott et al., 1994) and pro-opiomelanocortin (POMC; precursor of alpha-melano-stimulating hormone), ACTH and beta-endorphin from the anterior pituitary in ewes (Walsh et al., 1998). These compounds do not readily pass through the blood-brain-barrier to influence hypothalamic function, for the latter they have to be synthesised within the brain (Guillemin et al., 1977; Kastin et al., 1979).
- Each step in HPA axis activation can be mimicked by administering exogenous components: CRH infusion into ewe hypothalamus portal blood increases peripheral ACTH and cortisol (Naylor et al., 1990); ACTH i.v. injections increase cortisol (cows: Alam et al., 1986; ewes: Phogat et al., 1999a); whereas 10-day treatment of cows with betamethasone (a synthetic corticoid) suppresses plasma cortisol for up to 26 days and delays luteolysis probably by blocking prostaglandin F2-alpha release that is usually stimulated by follicular oestradiol (Dobson et al., 1987).

## Models to understand the interaction between HPA and HPO

Many acute stimuli models reveal reduced LH pulse frequency and amplitude as well as delay/block of the LH surge in cows and ewes by affecting difference phases of GnRH surge generation (Figure 2). For example, in rank order of stimulus severity:

- psycho-social models in ewes (social isolation, restraint, blindfolding and/or exposure to predatory cues; review: Ralph et al., 2016)
- transport in cows or ewes (Nanda et al., 1990a; Dobson et al., 1999),
- rapid reduction in plasma glucose by insulin injections (Saifullizam et al., 2010)
- exposure to LPS during the preovulatory period in cows and ewes (Battaglia et al.. 2000; Fergani et al., 2012).

To unravel changes that occur in these models, conventional approaches have been taken by endocrinologists to determine which part(s) of the activated HPA axis are responsible for disrupting normal function at specific levels of the HPO. While some studies investigate endocrine systems in cattle, many more use ewes that are more easily handled, less expensive to maintain, but closely mimic responses in cows. Different components of the HPO (as well as agonists or antagonists) have been administered to assess changes in responses during exposure to a variety of acute or chronic stimuli; and different HPA hormones have been assessed for their impact on HPO function. Moreover, a variety of reproductive states have been involved; for example, intact ewes in the breeding or non-breeding season, in luteal or follicular phases with or without (synthetic) hormonal manipulation, and short- or long-term ovariectomised animals with or without (synthetic) hormone replacement. There are difficulties in interpreting the responses in ovariectomised animals: the HPO itself adapts after ovariectomy, irrespective of any changes in HPA activity; selecting the correct replacement hormone in appropriate doses/patterns/duration affects responses; and the role of the animal’s own balancing systems are compromised (or often ignored during interpretation).

## Effect of increasing HPA activity on GnRH and LH pulsatile release

### Corticoids

In cows and ewes, during transport or treatment with betamethasone, the response to a single low dose of GnRH (to release LH in concentrations equivalent to a pulse) is lower (Dobson, 1987; Dobson et al., 1987). This action at pituitary level concurs with an infusion of cortisol (albeit for 30 h) that acts via glucocorticoid receptors to suppress LH pulse amplitude in ovariectomised ewes (review: Ralph et al., 2016). During transport-induced increases in endogenous cortisol, effects at the hypothalamus must also occur because the frequency of spontaneous LH pulses is lowered in ewes (review: Dobson and Smith, 2000b). Further proof of direct LPS action at the hypothalamus is revealed by a decrease in GnRH pulse frequency and amplitude in ewe portal blood (Battaglia et al., 1997, 2000).

### ACTH

In the follicular phase of intact ewes, LH responses to repeated challenges with GnRH are reduced by exogenous ACTH or transport (Phogat et al., 1999a, b). A second exposure to GnRH releases more LH than the first (self-priming effect) by providing more LH in a releasable form and increasing the number of GnRH receptors on the pituitary cell surface. *In vitro* perifusions of ewe pituitary slices show unequivocally that ACTH reduces the amount of LH released by repeated low dose GnRH; hence, a direct effect occurs at the pituitary and is enhanced with additional oestradiol (Phogat et al., 1997). Oestradiol also induces LH synthesis and GnRH receptor numbers, so any of the above processes may be interfered with to inhibit GnRH self-priming.

### CRH

Studies on the role of CRH in interrupting LH release in the ewe are initially confusing. Infusion of CRH peripherally or into the third ventricle of the brain in ovariectomised ewes either does not affect LH secretion, or causes an increase, in contrast to the rat or monkey in which CRH infusion results in prolonged inhibition of LH secretion (Clarke et al., 1990; Naylor et al., 1990). Now it is recognised that intra-cerebro-ventricular (i.c.v.) CRH increases GnRH/LH secretion only during periods of oestradiol negative-feedback (review: Smith et al., 2003a). Whereas, in the follicular positive-feedback phase, i.c.v. CRH suppresses LH pulse frequency (reversible by a CRH antagonist) and decreases both GnRH biosynthesis in the hypothalamus, and the number of GnRH-Receptors in the pituitary (Ciechanowska et al., 2018). There is also, however, direct suppression of CRH on LH released by a second low-dose GnRH challenge from the ewe pituitary *in vitro*, although the effects may have been mediated by *in vitro* pituitary release of ACTH (Smart, 1994). In rats, CRH reduces GnRH secretion *in vivo* or *in vitro* at hypothalamic level via opioid and catecholaminergic pathways (Rivier and Rivest, 1991).

## Effect of increasing HPA activity on surge secretion of GnRH and LH

### Corticoids

If intact cows or ewes are transported (for 25 min, 2 h or 8 h) just before an expected LH surge, the surge is delayed or totally blocked (Nanda et al., 1989b; Phogat et al., 1999b). The delaying effects on the LH surge are more marked if animals are transported close to the onset of an expected surge (review: Dobson and Smith, 1995). Delays could be due to progesterone from the adrenals because cows or ewes with plasma progesterone concentrations >0.5 ng/ml (endogenous or exogenous) do not have an LH surge in response to oestradiol (Nanda et al., 1988; Phogat et al 1999b). Ralph et al. (2016) conclude that the presence of oestradiol is necessary for cortisol to act at the level of the hypothalamus to interrupt GnRH/LH surges. However, during psychosocial stress, plasma cortisol increases but antagonism of glucocorticoid receptors does not block the effect of cortisol on LH suppression in ewes, indicating that there may be other factors acting in the hypothalamus to suppress GnRH secretion. Any actions of cortisol to inhibit GnRH are likely to be indirect because GnRH neurones in ewes do not contain glucocorticoid receptors, although pituitary LH cells do (Dufourny and Skinner 2002; Breen and Karsch, 2004).

### ACTH

If oestradiol is given to ewes in the follicular phase followed by 3 low doses of GnRH, an LH surge occurs within 40 h; if additional ACTH is given along with oestradiol, LH surges are blocked. Negative feedback of oestradiol is necessary to accumulate a readily-releasable LH pool, and it is possible that ACTH acts at pituitary level to prevent the replenishment of enough LH for a surge (Phogat et al., 1999a).

### CRH

During the ewe pre-ovulatory period, i.c.v. infusion of CRH suppresses the LH surge causing prolonged oestrus cycles, via effects at the hypothalamus and pituitary (Polkowska and Przekop, 1997; Ciechanowska et al., 2018).

### Gonadotrophin inhibiting hormone (GnIH)

This compound, first identified in birds, may be a possible mediator of the effects of increased HPA activity on reproduction. A mammalian homologue of GnIH, RF-amide-related peptide-3 (RFRP-3), suppresses GnRH/LH secretion in ewes but immunohistochemistry and *in situ* hybridisation studies on the hypothalamus of long-term ovariectomised ewes fail to show an increase in GnIH activity after psychosocial stress (review: Ralph et al., 2016).

### Opioids

An early series of cow studies using exogenous opioid agonists and antagonists hint that there is opioid suppression of LH release until hour(s) before the LH surge, that is then lifted and the LH surge occurs; however, the suppression is extended by transport (blocking/delaying the LH surge), although the latter is not reversed by one injection of opioid antagonist (Nanda et al., 1989b, 1990b, 1991b, 1992a). Recent work shows that reversal of stimulus-induced LH surge suppression requires several hours of opioid antagonist infusion (Dobson et al., 2020b).

## Effect of increasing HPA activity on peripheral FSH concentrations

From the scarce literature available, mid-cycle FSH is increased by HPA stimulation but this is probably due to a reciprocal reduction in follicular oestradiol after suppression of GnRH/LH pulses. Late follicular phase surges of FSH are delayed in parallel with LH surges (Roth et al., 2000; Battaglia et al., 2000).

## Models for in-depth studies

With the back-ground of all our above studies, we have recently focused on two main paradigms:

- a chronic spontaneously-occurring model comparing clinically compromised cows (lame, high somatic cell count, low body condition score) with their ‘normal’ herd-mates might reveal ways in which cows utilise coping trade-off strategies, and
- an acute ewe model with or without insulin or LPS treatment that has focused on how these trade-offs may be controlled by neurotransmitters within the brain.

## Chronic models of milking cows that are compromised by production diseases

Behavioural observations on-farm reveal that lame cows have lie for longer, spend less time expressing oestrus, have a lower bite rate at pasture and lower body condition scores – all situations that compromise fertility (Walker et al., 2008a). Also, the follicular phase is shorter in cows with high somatic cell count (SCC), and more cows with high SCC and lameness fail to ovulate (Morris et al., 2009, 2013). Further synergistic effects are revealed in cows with 0, 1 or 2 ‘severe’ production diseases that have intervals from calving to the first luteal phase of 31, 44 and 54 days, respectively (Peake et al., 2011).

Spontaneous LH pulse frequency and plasma oestradiol concentrations are lower in lame non-ovulating cows compared to those that do ovulate (Morris et al., 2011). However, the situation is not binary: from 30 to 80 days post-partum, there is a graded effect that ranges from ~ 30% lame cows with no ovarian activity, another ~ 20% fail to express oestrus or ovulate a low oestrogenic follicle; but in 50% cows, many reproductive parameters are unaffected by lameness (Morris et al., 2011). It is not yet clear how some lame cows cope whereas others shut-down to achieve this graded trade-off.

Turning to oestrus behaviour, continuous visual monitoring for all signs of oestrus in cows (uncompromised by clinical conditions) reveals a precise sequence of events (Dobson et al., 2018). Sniffing other cows is followed by active behaviours of mounting another cow and not accepting a mount, as well as the passive behaviours of being sniffed and standing-to-be-mounted (STBM) by another cow. Chin resting occurs before refusing a mount and STBM. All these behaviours occur in the reverse order after the last STBM. Such distinct behavioural sequences are probably controlled by changes in peripheral progesterone and oestradiol concentrations, as well as by subtle independent mechanisms via pheromones in differing concentrations and/or divergent composition.

Lameness does not affect the above sequence or overall duration of oestrus (although the intensity is reduced; Walker et al., 2008a, 2010). Despite the pain, lame cows are willing to be involved in some sexual activity but the frequency of their own mounting is minimised as well as the duration of being attractive to others (attempts at being mounted). Lame cows also exhibit fewer chin rests – a behaviour that solicits mounting from herd-mates, as well as testing whether it is worth expending energy and/or enduring pain to mount others (another trade-off). So, lame cows may be less attractive to others by emitting poorer quantity/quality of sexual pheromones or even ‘stress-related’ pheromones, as cows can perceive increased stress in herd-mates by olfactory cues (Boissy et al., 1998). The quantity of the chemical signals may not be as important as the interpretation of chemical messages (i.e., the reward mechanisms).

From a practical stand-point, the efficiency of activity monitoring devices (neck collars and pedometers) is reduced by lameness, low BCS or high milk yield no doubt due to the above effects on oestrus intensity (Holman et al., 2011). As mentioned before, only 74% of all potential oestrus periods (episodes of low progesterone) are identified by combining information from activity monitoring devices and farm staff observations. The endocrine environment is prepared for expressing oestrus but the cows ‘choose’ not to. Extending these observations, Williams et al. (2018) note that total motor activity detected by the devices is lower in lame cows, and when potential oestrus events (low milk progesterone value between two high values) are not recognised by both devices, progesterone values are slightly higher at the potential oestrus (0.043 *versus* 0.029 ng/ml), contrary to luteal values that are markedly lower in lame cows (1.3 *versus* 0.7 ng/ml; Walker et al., 2008a, 2010).

Overall, studying these chronic conditions on-farm has high-lighted the fact that cows are operating a trade-off between their environment and reproductive efficiency. A delicate knife-edge balance operates – some stimuli do not have a deleterious effect and can be coped with by some animals, but not all. The severity of the environment dictates outcomes, as do combinations of production diseases. A significant way of coping involves modifying oestrus behaviour to avoid getting pregnant, as well as reducing the incidence of ovulation. Interestingly, similarities exist between chronic situations and acute experimental models: LH pulse frequency is decreased, oestradiol concentrations are consequently reduced and subsequent luteal progesterone values are lower. But exactly how do animals modify these vital parts of reproductive function to bring about the trade-off?

## Role of neurotransmitters in ewes subjected to transport, insulin or LPS (acute models)

**Figure 6:**
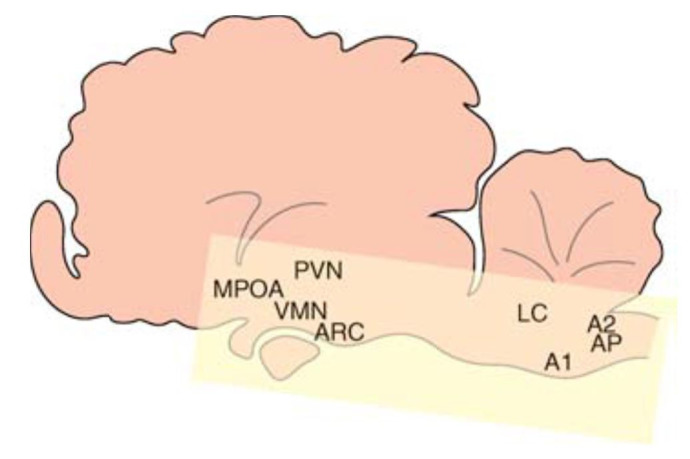
Location of neuronal nuclei in the ewe brain that regulate GnRH and corticotrophin-releasing hormone and arginine vasopressin secretion, indicating the spatial relationship between regions of the hypothalamus (medial preoptic area (mPOA), ventral medial nucleus (VMN) and arcuate nucleus (ARC) of the medial basal hypothalamus)) and the brain stem (A1 and A2 regions, the area postrema (AP) and the locus coeruleus (LC)). PVN: paraventricular nucleus. Adapted from Dobson et al. (2003); reproduced with permission.

There is a delicate balance between positive and negative influences of steroids and neurotransmitters in various locations in the brain (Figure 6). The ultimate goal is to respond to stimuli in the environment in order to regulate the pattern of GnRH secretion into hypophyseal portal blood.

When a stimulus is perceived, noradrenaline-neuropeptide Y neurones are activated in the brain stem (A1 and A2 regions, the area postrema (AP) and the locus coeruleus (LC) with on-going synaptic contacts between these cells and CRH/AVP cell bodies in the hypothalamic paraventricular nucleus (PVN), possibly also with links via the medial pre-optic area (mPOA; Dobson et al., 2003). As a result, CRH/AVP are transferred along neurones to the median eminence with subsequent release into the hypophyseal portal system, and consequent stimulation of ACTH secretion from the pituitary. In the PVN, CRH/AVP cells contain glucocorticoid receptors that exert strong and quick negative-feedback, an essential process to prevent prolongation of enhanced glucose metabolism, altered vascular dynamics and dysfunctional immune responses.

### Effects of neuronal agents on HPO and HPA activity

Sedation induced by sodium pentobarbitone reduces the magnitude or totally blocks the LH surge in cyclic and oestradiol-treated ewes or cows (Dobson and Ward, 1977; Nanda et al., 1992b). The expected LH surge in late follicular phase ewes or oestradiol-treated anoestrous ewes is also totally suppressed by the alpha-adrenergic blocker, phenoybenzamine, indicating an adrenergic role in initiating the LH surge (Narayana and Dobson, 1979).

Pentobarbitone, diazepam (gamma-amino-butyric acid (GABA) agonist), or xylazine (alpha-2 adrenergic agonist) all immediately decrease basal, as well as transport-induced increases in plasma cortisol, glucose, respiration rate and heart rate (Sanhouri et al., 1991a, c, 1992). However, xylazine does not affect the cortisol response to exogenous CRH, indicating a mechanism mediated through the hypothalamus (Sanhouri et al., 1992). In contrast, an alpha-1 adrenergic antagonist or a beta-adrenergic blocker do not lower cortisol responses to transport (Sanhouri et al., 1991b).

All these systemically administered agents could suppress afferent sensory input to the brain (perception) or interfere with efferent responses within the brain. Concerning the latter, the paraventricular nucleus (PVN) has alpha-1 adrenergic receptors, is well innervated by adrenergic fibres, and administration of an alpha-1 adrenergic agonist directly into the third ventricle of the brain increases peripheral ACTH and cortisol concentrations (Liu et al., 1991; review: Dobson et al., 2003).

### In vitro inter-relationships between neurotransmitters, CRH/AVP or GnRH activity in ewes

Perifusions of ewe hypothalamus slices examine direct effects of steroids and neurotransmitters. Exposure to oestradiol increases basal release of AVP (Ghuman et al., 2006), and alpha-1-adrenoreceptor agonists increase AVP secretion (concurring with the above *in vivo* studies) - this response is further enhanced in the presence of oestradiol (Ghuman et al., 2008a). Using the same perifusion system with agonists and antagonists to GABA, Ghuman et al. (2007) conclude that basal AVP release is under GABA-B inhibition, and this negative effect is enhanced by oestradiol.

Concerning *in vitro* GnRH secretion in ewes, oestradiol again has considerable impact via adrenergic and GABA-ergic influence. Basal GnRH increases in the presence of oestradiol (Ghuman et al., 2006) and an adrenergic agonist increases release of GnRH that is prolonged by oestradiol (Ghuman et al., 2008b). In contrast, a GABA-A antagonist results in greater GnRH secretion that is higher in the absence of oestradiol (Ghuman et al., 2008c) concurring with *in vivo* studies (Scott and Clarke, 1993). It is suggested that higher concentrations of oestradiol eventually decrease GABA turnover which in turn facilitates activation of GnRH neurones. This is supported by the marked decrease in GABA tone just before the GnRH/LH surge co-incident with high oestradiol concentrations in ewes (Robinson et al., 1991). Furthermore, in the presence of low oestradiol concentrations, noradrenaline (NA) stimulates GABA to suppress GnRH release, but this effect is decoupled by the high oestradiol values of the pre-ovulatory period (by currently unknown mechanisms).

### In vivo inter-relationships between neurotransmitters, CRH/AVP and GnRH in ewes

**Figure 7:**
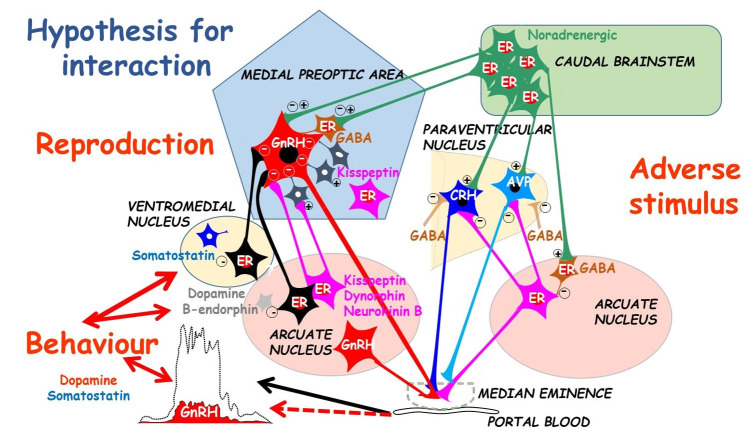
Diagram indicating possible inter-action between neurones involved in GnRH/behaviour disruption following adverse stimuli imposed in the late follicular phase of the ewe. Noradrenergic cells in the brain stem project to both the paraventricular nucleus (PVN) and the medial preoptic area (mPOA). Adverse stimuli activate CRH/AVP neurones in the PVN. Changes in activity of beta-endorphin and dynorphin neurones in the arcuate nucleus (ARC) influence PVN and mPOA output. In the ARC, the activities of oestradiol receptor (ER) neurones (probably kisspeptin/dynorphin/neurokinin B; KNDy cells) are altered by adverse stimuli, as are ER cells in the ventromedial nucleus (VMN). In the median eminence, CRH, but not AVP, terminals and KNDy terminals are in close contact with GnRH terminals providing another site for the disruption of GnRH release. Positive or negative effects at cell bodies are circled. Adapted from original drawing by SPS Ghuman (Dobson et al. (2012); reproduced with permission).

Snapshots of inter-relationships between/within brain and hypothalamic nuclei are obtained by immuno-histochemistry (Figure 7). In the late follicular phase of ewes, noradrenergic terminals are in close contact with many CRH and AVP cell bodies in the PVN but not with beta-endorphin cell bodies in the arcuate nucleus (ARC). Furthermore, GABA terminals are close to CRH, but not AVP, cell bodies in the PVN, as well as beta-endorphin cells in the ARC (Ghuman et al., 2010, 2011). Although CRH, AVP and beta-endorphin terminals are seen in the mPOA, there are no direct contacts with GnRH cell bodies in this area. Within the median eminence, abundant CRH (but no AVP) terminals are close to GnRH cell terminals in the external zone; whereas, beta-endorphin and dynorphin cell bodies and terminals are in the internal zone (Ghuman et al., 2011; Clarke, 2015). The presence of c-Fos (an early gene activation marker) indicates which cells are currently active: after *in vivo* insulin treatment in ewes, the number of activated noradrenergic neurones in the caudal brainstem increases markedly, along with significant activation of CRH and AVP neurones in the PVN. Despite a general increase in activated neurones in the ARC, the number of activated oestradiol receptor alpha (ER) neurones is reduced by insulin treatment (Ghuman et al., 2011).

All this neuroanatomical evidence supports the hypothesis that brainstem noradrenergic and hypothalamic GABA neurones are important in modulating the activity of CRH and AVP neurones in the PVN, and beta-endorphin neurones in the ARC. These PVN and ARC neurones may also activate inter-neurones to influence GnRH cell bodies in the mPOA, whereas the median eminence is also a major site for direct modulation of GnRH release by CRH terminals (review: Dobson et al., 2003).

### Further in vivo studies on regulation of GnRH in intact control ewes

In ewes, when peripheral progesterone concentrations have decreased and oestradiol concentrations reach a threshold value (at least 6–7 h before the expected LH surge onset), complex interactions occur particularly between/within the brain stem and hypothalamus. The hypothalamic mPOA is the main location of GnRH cells and (in response to modulation from the brain stem, ARC, VMN and PVN) the transfer of GnRH proceeds along axons to the median eminence for secretion into portal blood, and onward to the pituitary to release LH (Figure 7).

**Figure 8:**
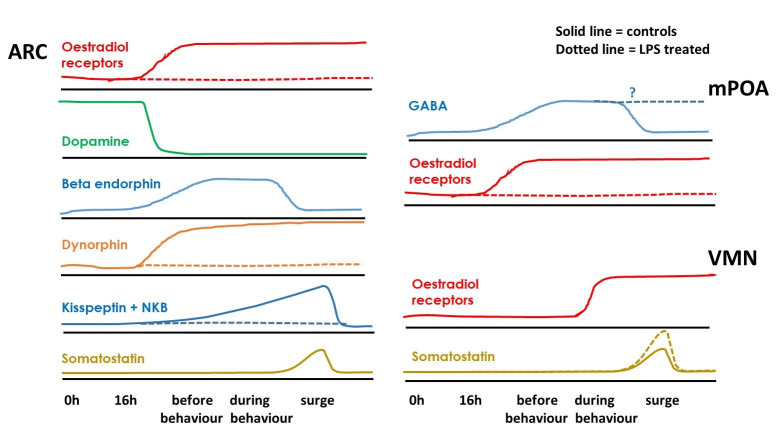
Diagram of neurotransmitter cell influence (i.e., % cells c-Fos activated) in the hypothalamic arcuate nucleus (ARC), medial pro-optic area (mPOA) and ventro-medial nucleus (VMN) at different times during follicular phase of intact ewes with (dotted line) or without treatment with lipopolysaccharide toxin (LPS) at 28 hours; LH surge occurs at 40 hours. No dotted line indicates no difference from controls. Adapted from Fergani et al. (2014b, 2017); and Robinson et al. (1991).

There are no adrenergic cell bodies in the hypothalamus but axons extending from noradrenergic cells in the brainstem are in close contact with GnRH cell bodies in the ewe mPOA (Clarke et al., 2006). There are also reciprocal links between the ARC and VMN, as well as significant connections to both areas from the PVN (Qi et al., 2008). Regarding neurotransmitters involved in these links, many neurones within the ARC and VMN contain ER, beta-endorphin, dopamine or somatostatin (SST); but existence of any co-localisations in the ewe require more studies (Jansen et al., 1997; Elmquist 2001; Qi et al., 2008; Fergani et al., 2015). Nevertheless, GnRH release is modulated by a specific sequence of interactions between the mPOA, ARC and the VMN. The number of activated ER neurones increases gradually in the mPOA throughout the ewe follicular phase, reaching maximum prior to the surge onset (Figure 8); however, in the ARC, ER activation increases just before the onset of sexual behaviour and remains high throughout the GnRH/LH surge; whereas, in the VMN activation only exceeds base-line during sexual behaviour. Notably, activation of ER cells is maximal during the LH surge in all these areas, substantiating the role of oestradiol-positive feedback in GnRH surge secretion (Fergani et al., 2014b).

Further cell types within the ARC are also activated at varying times before the GnRH/LH surge in ovary-intact ewes (Figure 8; Fergani et al., 2017). Specifically, activation of dopamine neurones is initially high, but decreases just before behaviour onset, whereas activation of beta-endorphin cells increases in the mid-follicular phase, then decreases a few hours later during the surge.

Also in the ARC, ‘**KNDy**’ cells contain ER and neuropeptides, the latter being both stimulatory (**K**isspeptin, **N**eurokinin B (NKB)) and inhibitory (**Dy**norphin) to keep GnRH secretion tightly regulated. However, the overall balance of different neuropeptides within KNDy cells varies throughout the ewe follicular phase. Initially, there is a shift of the net balance towards inhibitory dynorphin before behaviour starts, followed by a swing towards excitatory kisspeptin and NKB after the influence of increasing oestradiol concentrations during the surge (Fergani et al., 2017). Also, some cells in the ARC and VMN contain SST and these are maximally activated during the LH surge (Figure 8; Fergani et al., 2014b). SST may be involved in GnRH/LH surge termination because it is a very potent inhibitor of electrical excitability of GnRH neurones, but it is also implicated in control of sexual behaviour (see later).

There are claims that the KNDy neuronal network is the true controller of the ewe reproductive system, with the GnRH neurones being the primary output signal from the brain (Scott et al., 2018). This is mainly because KNDy cells constitute the GnRH pulse generator: the signal to stimulate a GnRH pulse is initiated by NKB activity within the KNDy neurone network, while dynorphin stops kisspeptin release from the KNDy neurones thus ending a pulse (Lehman et al., 2010). The majority of KNDy cells are in the ARC and mPOA with a few in the VMN. Approximately 50% KNDy neurones in the mPOA have ER, whereas virtually all kisspeptin cells in the ARC have ER and progesterone receptors. Intriguingly, KNDy cells are inhibited by low doses of progesterone and oestradiol, but stimulated by high doses of oestradiol. KNDy cells also receive synaptic input from neurones that contain glutamate, dopamine, and POMC. Thus, KNDy neurones integrate a lot of information about the internal and external environment of animals, and then act on GnRH cell bodies and dendrites in the ARC and mPOA, as well as the median eminence, to influence the release of GnRH into hypophyseal portal blood (Scott et al., 2018).

### Effects of acute LPS treatment on ewe hypothalamic neurotransmitters

Treatment of ewes with LPS increases c-Fos and CRH mRNA within the PVN, increases secretion of CRH into portal blood, and increases CRH-Receptor (CRH-R) activity in the lower part of the ARC and median eminence (Vellucci and Parrott, 1996; Battaglia et al., 1998; Breen and Karsch, 2004; Fergani et al., 2013). At the same time, the LH surge is delayed for as long as the oestradiol signal is disrupted (Fergani et al., 2012). This is accompanied by reduced activation of ER in the mPOA, and ER, dynorphin and kisspeptin cells in the ARC, but possible enhanced SST cell activation in the VMN (Figure 8; Fergani et al., 2013, 2014b, 2017). However, LPS has no effect on the activation of dopamine, β-endorphin or SST cells in the ARC raising the possibility that these cell types are only permissive in the surge induction process in ewes.

Glucocorticoid (cortisol) receptors do not exist on GnRH neurones but are co-localised with progesterone and oestradiol receptors in the mPOA and ARC, possibly in KNDy cells, and it is by this indirect pathway that cortisol signals are transmitted to ewe GnRH neurones (Dufourny and Skinner, 2002; Goodman et al., 2007; review: Lehman et al., 2010; Fergani et al., 2017). There is also an abundance of CRH-Rs in the median eminence (Ghuman et al., 2010). Therefore, there are two possible (simultaneous, therefore fail-safe?) pathways for CRH suppression of GnRH release via CRH-Rs: one being the regulation of kisspeptin/dynorphin and other cell types in the ARC, and the other being the direct association of CRH and GnRH cell terminals in the median eminence.

### Effects of acute insulin treatment on ewe hypothalamic neurotransmitters

A CRH antagonist does not prevent the inhibitory effect of insulin on LH pulses in ovariectomised ewes (Clarke et al., 1990). Indeed, contrary to expectation, CRH-R cell activation in the ewe ARC and the median eminence remains unaltered after insulin treatment, and there is immediate increased activation of kisspeptin cells in the ARC but not in the mPOA (Fergani et al., 2014a). This may be a result of insulin activating directly (on kisspeptin neurones) or indirectly (via POMC/beta-endorphin neurone activation). Insulin stimulates SST activation in the ARC of all insulin-treated ewes but this is part of the glucose-sensing mechanism (i.e., stimulus specific), and there is no ER activation in this region. Thus, a reduction in stimulatory kisspeptin cell activation is not part of the GnRH/LH inhibiting mechanism after PVN activation by insulin. However, there is increased SST activation in the VMN along with decreased ER activation in the mPOA: patterns similar to those after LPS indicating a common pathway (Fergani et al., 2015).

## Characteristics of oestrus behaviour in cows and ewes

Now that we know more about neurotransmitter and steroid regulation of the HPO, it is worth returning to consider in detail how oestrus behaviour is controlled. Beach (1976) divides ewe pre-copulatory (sexual) behaviour into three components: attractivity, proceptivity and receptivity. Starting with oestrous females being searched for by males (attractivity), the male and female then come near each other, and the male eventually closely noses the female’s perineum (proceptivity), transferring both olfactory and gustatory information from the female to the male. A series of courtship behaviours follow including tail fanning (by which the ewe aerially disseminates perineal pheromones), vocalisation, the male resting his chin on the female’s back, nudging and pawing of the female (by which the male tests the willingness of the female to be mounted, i.e., in response to male pheromones, the female does not move away). Eventually there is mounting (receptivity), coincident with onset of the LH surge (Fergani et al., 2012). Sight, sound and smell are all important for contacts between oestrous females and males, but smell is imperative to achieve successful mounting in ewes (Fletcher and Lindsay, 1968). The initial sequential build-up of individual but very different behaviours is understandable as females need to be near males for nosing to take place, followed by nudging to test if the female is ready to immobilise, then finally the male mounts the female. However, the reason for the reverse sequential loss of these behaviours, rather than an abrupt cessation of all behaviours, is less clear. In all female groups, especially cows, a herd-mate will take on the role of male but a similar sequence of behaviours occurs (Walker et al., 2008a)

## Effects of chronic or acute stimuli on oestrus behaviour in cows and ewes

Lameness in milking cows does not affect the incidence of oestrus but does reduce oestrus intensity and is associated with lower luteal milk progesterone values prior to oestrus (Walker et al., 2008b). Specifically, the period when herd-mates attempt to mount a lame cow is shorter, and lame cows are mounted less frequently (i.e., are less attractive; Walker et al., 2010). Cows with high SCC or sub-clinical ketosis also have a less intense oestrus with lower oestrus scores (Morris et al., 2013; Rutherford et al., 2016).

**Figure 9:**
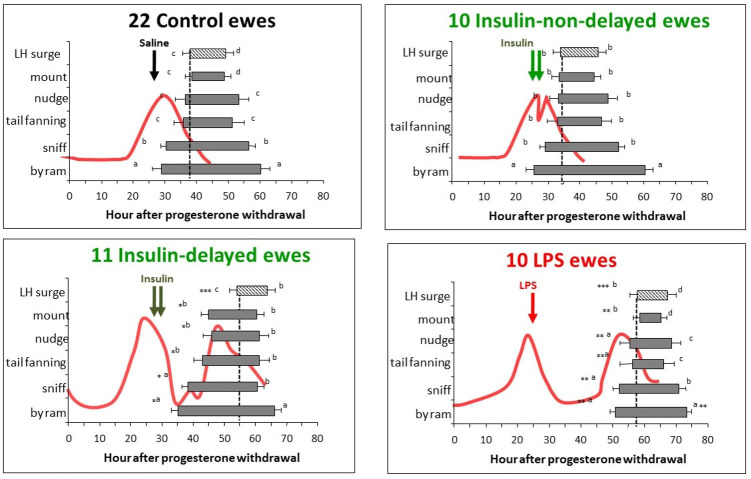
Mean (± SEM) hours from first to last display of different oestrus behaviours after progesterone withdrawal in 22 control ewes, 21 ewes injected with 4 IU/kg insulin (non-delayed n=10, delayed n=11) at 28 and 30 h, and 10 ewes injected with 100 ng/kg LPS at 28 h after progesterone withdrawal. Also shown: mean plasma oestradiol concentrations (red line) and timing of the LH surge (with onset indicated by dashed vertical line). Within each panel, differences between the onsets of each behaviour are indicated by different letters at each end of each bar, respectively (P<0.05); differences between the duration of each behaviour are also indicated by the letters at the end of each bar (P<0.02). Differences in the timing of onset between panels are indicated with asterisks. Time of treatment is indicated with the arrows. *P<0.05 compared to controls and insulin-non-delayed groups, **P<0.001 compared to control and both insulin groups, ***P<0.001 compared to controls and insulin-non-delayed groups. ****P<0.05 compared to controls and insulin subgroups. Adapted from Fergani et al. (2012); reproduced with permission.

In ewes, insulin or LPS treatment in the follicular phase decreases peripheral oestradiol concentrations and delays the LH surge (Saifullizam et al., 2010; Fergani et al., 2012). All behaviours are delayed more or less together as a group, while durations and most frequencies are not affected, indicating that they may all have a common regulating factor, probably oestradiol. However, as they begin and end sequentially, each must also incorporate distinct controlling mechanisms. Furthermore, oestradiol at differing concentrations is able to separate the initiation of behaviour and the preovulatory LH surge (Figure 9; Saifullizam et al., 2010; Fergani et al., 2012).

In cows and ewes, a period of alternating high/low peripheral progesterone concentrations, followed by oestradiol is the primary ‘trigger’ for the onset of sexual behaviour and the GnRH/LH surge, although the threshold concentration for the induction of sexual behaviour may be lower than that for the GnRH/LH surge; therefore, the triggering signals may be different in ewes (Caraty et al., 2002; Ben Said et al., 2007). Insulin or LPS treatment of ewes causes a slight increase in progesterone concentrations and this subtle change may be part of the mechanism by which sexual behaviours are disrupted although it is unlikely that the progesterone increment is the sole mediator because the effect is not reversed by the progestin/glucocorticoid receptor antagonist RU486 (Dobson and Smith, 1998; Dobson et al., 2020b).

## Role of neurotransmitters in the control of ewe oestrus behaviour

*Somatostatin:* In the ewe, the ARC and VMN constitute major sites for oestradiol to regulate the induction of sexual behaviour (and the preovulatory GnRH surge; Blache et al., 1991; Caraty et al., 1998). Indeed, there is a positive correlation between activated ER cells in the VMN and peripheral oestradiol concentrations (but not progesterone). Furthermore, there is a distinct temporal pattern of ER cell activation that begins in the ARC and mPOA at least 6–7 h before the onset of ewe sexual behaviour, but only later extends to the VMN during behaviour (Figure 8; Fergani et al., 2014b). Moreover, the ARC and VMN have subpopulations of cells co-localising ER and SST (Scanlan et al., 2003; Herbison, 1995). These SST cells may mediate in the ER control of ewe sexual behaviour (and the preovulatory GnRH surge).

Indeed, intact ewes treated with LPS during the late follicular phase did not express sexual behaviour, and this was accompanied by the failure of ER cell activation in the ARC and changes in SST cell activation in the VMN (Fergani et al., 2014b).

*Noradrenaline (NA):* Activation of ER neurones in the VMN increases 10-fold during ewe sexual behaviour (Fergani et al., 2014b) co-incident with an increase in noradrenaline (NA) in extracellular fluid of the mediobasal hypothalamus (MBH; containing the ARC and VMN; Fabre-Nys et al., 1997). This NA probably arrives via axons from NA cell bodies in the ewe brain stem where there is co-localisation with ERs (Ghuman et al., 2008a). More detailed investigations to determine whether SST cells receive input from suppressive dopamine and/or excitatory NA cells over this period are awaited to explain the delay in ER cell activation in the VMN.

*Dopamine*: This neurotransmitter is involved in the control of ewe sexual behaviour (Fabre-Nys and Gelez, 2007). Dopamine neurones in the ARC are maximally activated in the early follicular phase but this decreases markedly just before signs of oestrus begin in the ewe (in a reciprocal pattern to ER activation in the VMN; Fergani et al., 2017). This consistent with initially high extra-cellular concentrations of dopamine in the MBH, followed by a sharp decrease preceding the onset of ewe sexual behaviour (Fabre-Nys and Gelez, 2007).

However, ewes treated with LPS do not exhibit signs of sexual behaviour but dopamine cell activation in the ARC is not affected, indicating that this pathway may be permissive but not indispensable for the initiation of oestrus in ewes (Figure 8; Fergani et al., 2017).

**Figure 10:**
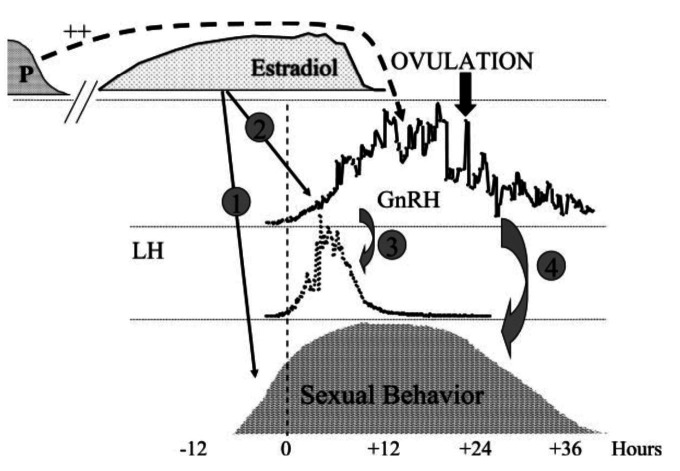
In the ewe at luteolysis, plasma progesterone decreases and oestradiol increases. Oestrus behaviour is initiated (1) when oestradiol reaches a threshold and remains high for 6–10 h. Simultaneously, oestradiol also induces a surge of GnRH in portal blood (2), and LH in the periphery (3). Co-incident with the LH surge, oestradiol declines to basal concentrations while GnRH secretion stays high for an additional 24–36 h, while oestrus behaviour continues (4). One role of progesterone priming (P) is to increase the magnitude of the GnRH surge (broken arrow). Ovulation (black arrow) is precisely timed occurring 22–26 h after the LH surge. Adapted from Caraty et al. (2002); reproduced with permission.

*GnRH:* Sexual behaviour is enhanced by peripheral or central administration of GnRH in gonadectomised and/or hypophysectomised rodents, eliminating the possibility that GnRH itself mediates behaviour indirectly by increasing levels of gonadal steroids (Schiml and Rissman, 2000). Evidence for exogenous GnRH is not so strong in ewes, but at the end of the follicular phase there is a synchronous surge release of GnRH and LH secretion, with LH returning to baseline after ~10 h, whereas GnRH secretion remains elevated for another 24 h co-incident with the maintenance of receptive behaviour (Figure 10; Caraty et al., 2002). In the ewe, progesterone priming is associated with increases in ER in the ARC/VMN, and greatly increases the magnitude of the GnRH surge; and subsequent sequential actions of oestradiol and GnRH ensure full expression of oestrus behaviour, with extended preovulatory GnRH secretion prolonging receptivity after oestradiol has disappeared from the peripheral circulation. Thus, Caraty et al. (2002) concluded that GnRH has a facilitatory role in the control of sexual behaviour in the ewe.

Suppressed intensities of sexual behaviours after LPS are associated with lower oestradiol concentrations in peripheral plasma, partially by directly affecting production in the ovarian follicular granulosa (Price et al., 2013), as well as by decreasing GnRH concentrations in hypophyseal blood (Battaglia et al., 1997; Fergani et al., 2012).

## Role of the pheromonal system

Pheromones are used for social communication between animals, male or female. Olfactory substances emitted by an individual induce genetically pre-programmed behavioural and/or physiological responses in recipients (Johnston and Bronson, 1982; Dulac and Wagner, 2006). For example, when vulval skin gland secretions are presented to a bull in the absence of a cow/heifer, positive responses are observed indicating that vulval skin glands may be a specialized site for the production or concentration of pheromones (Rivard and Klemm, 1989). After pheromones have been produced and dispersed, in this other instance by males, the information is received by vomeronasal receptors in the ewe vomeronasal organ (VNO) and then transmitted to the accessory olfactory bulb (AOB) via the vomeronasal nerves, thereby prompting the neuroendocrine system of the recipient ewe to display signs of oestrus (Kratzing, 1971).

However, while there is intense activity of c-Fos and ELOVL5 (a putative pheromone synthetic enzyme; Momozawa et al., 2007) in the vulva of control and LPS treated ewes, a reduction in ram behaviour towards ewes was not accompanied by quantitative changes c-Fos or ELOVL5 in the ewe vulva, but subtle qualitative differences in individual specific compounds (attraction pheromones) remain an option (Dobson et al., 2020a). Furthermore, having been treated with LPS to inhibit sexual behaviour, there were no differences in the ewe vomeronasal organ with respect to cell type or intensity of c-Fos activity, pheromone receptors, or olfactory marker protein, dismissing the vomeronasal organ as a major site involved in the suppression of sexual behaviour (Dobson et al., 2020c). Indeed, along with other hypothalamic nuclei, the bed nucleus of the stria terminalis (BNST) in the ewe is activated just before the expected onset of oestrus and, based on current neuroanatomical data, this activated nucleus is involved in the transmission of pheromonal signals from the amygdala, as well as transferring information via projections to the mPOA where most GnRH cells are located. However, LPS treatment is not associated with any changes in BNST activation (Fergani et al., 2013), so other parts of the ewe hypothalamus are more likely location(s) of action.

## In conclusion

There is still a lot we do not know/understand. The above evidence reviews current information regarding interactions between the environment and fertility via the HPA and HPO, especially focusing on control exerted via KNDy and GnRH cells. However, we still do not know exactly how those cells are governed in any species, particularly co-localisation and/or interdependence of ER, beta-endorphin, dopamine or SST in cells within the mPOA, ARC and VMN. It is also necessary to know more about the expression of oestrus behaviour: is there any direct involvement of GnRH? What is the precise nature of female pheromones, and how/when are they produced? What are the internal mechanisms involved in the ‘reward’ system that stimulates females to express oestrus?

Returning to the ‘random’ observations in the opening section outlining examples of trade-offs: Wildebeest migrate large distances to find food and they are very efficient at reproduction – these major trade-offs arise because they are not exploited by humans for meat/milk production. When we domesticate animals for our own use, it is our responsibility to provide the best possible environment (housing, lying/walking areas, etc) otherwise animals trade-off with lower production, less intense oestrus behaviour, and impaired fertility. The incidence of (sub) clinical problems around calving should be minimalised, but why make cows calve so frequently? Lactation persistency is enhanced by increasing milking frequency, feeding more concentrates during declining lactation, and by genetic selection. Pregnancy rates in later lactation are similar to those soon after calving, and later re-breeding of high-yielding cows improves profitability (Dobson et al., 2007). Avoiding life-time peri-parturient problems by managing persistent lactations could be a worthy trade-off on both welfare and economic terms.

Trying to understanding how cows and ewes achieve trade-offs is still on-going. If the environment is not ideal for passing genes on to the next generation, it is essential that animals can temporarily delay conception (often by avoiding expression of oestrus behaviour) until conditions improve. Ultimately, those animals that do develop coping strategies to overcome adverse stimuli, are the ones with the genes that are valuable to achieve trade-offs. From the fore-going discussion, it is clear that survival of the species/individual is so important that the strategy does not depend on a single factor, but is made fail-safe by being multi-factorial, achieved through a complex interplay among excitatory and inhibitory neuronal and hormonal signals that converge on hypothalamic neurones responsible for secretion of GnRH. Understanding the mechanisms of this interplay is of paramount importance for the efficiency of both sexual behaviour and subsequent fertility.

We know that cows and ewes do exert fertility trade-offs, but we need to be sure we are using the right criteria to monitor their impact, e.g., meaningful welfare indices are required, and assessing output per animal/rumen rather than per kg milk/meat may be more appropriate in view of the reverse impact exerted by ruminants upon the environment (green-house gases, waste product disposal, etc.). Domestication needs to be as efficient as possible both economically and environmentally. However, while optimising the inter-relationship between animals and the environment, caution is urged against the camouflage use of drugs/hormones/feed additives/intricate technologies (Higgins et al., 2013). In the long term, a better strategy is to get animals and environment in a more harmonious balance.

Finally, there is one phenomenon that remains totally inexplicable. How/why do some animals display oestrus during pregnancy? In one study, ~ 5% dairy cows displayed oestrus (sniffed and mounted by herd-mates) at least once at varying times throughout pregnancy – some even stood willingly to be mounted and mated by a bull. However, dried samples of vaginal mucus did not form characteristic fern patterns; and plasma oestradiol concentrations were similar to non-pregnant oestrus herd-mates, whereas plasma progesterone values were all > 1.5 ng/ml when expressing sexual behaviour (Thomas and Dobson, 1989). How/why?

## Abbreviations

ACTH: adreno-corticotrophin hormone

AI: artificial insemination

AOB: accessory olfactory bulb

AP: area postrema

ARC: arcuate nucleus

AVP: vasopressin

BCS: body condition score

BHB: 3-beta-hydroxy-butyrate

BNST: bed nucleus of the stria terminalis

CRH: corticotrophin releasing hormone

CRH-R: corticotrophin releasing hormone receptor

DMI: dry matter intake

ELOVL5: Elongation of very long chain fatty acids protein 5

ER: oestradiol receptor alpha

FSH: follicle stimulating hormone

GABA: gamma-amino-butyric acid

GnIH: gonadotrophin inhibiting hormone

GnRH: gonadotrophin releasing hormone

HPA: hypothalamus-pituitary-adrenal axis

HPO: hypothalamus-pituitary-ovarian axis

i.c.v.: Intra-cerebro-ventricular

IU: international units

KNDy: kisspeptin, Neurokinin B and Dynorphin

LC: locus coeruleus

LH: luteinising hormone

LO: left ovary

LPS: lipopolysaccharide toxin from *E coli*

MBH: mediobasal hypothalamus (containing the ARC and VMN)

mPOA: medial pre-optic area

mRNA: messenger ribonucleic acid

NA: noradrenaline

NEFA: non-esterified fatty acid

NKB: neurokinin B

POMC: pro-opiomelanocortin

PRID: progesterone releasing intravaginal device

PVN: paraventricular nucleus

RFM: retained fetal membranes

RFRP-3: RF-amide-related peptide-3

RO: right ovary

SCC: somatic cell count

SEM: standard error of the mean

SST: somatostatin

STBM: standing-to-be-mounted

VNO: vomeronasal organ
